# 5-FU resistant EMT-like pancreatic cancer cells are hypersensitive to photochemical internalization of the novel endoglin-targeting immunotoxin CD105-saporin

**DOI:** 10.1186/s13046-017-0662-6

**Published:** 2017-12-19

**Authors:** Kaja Lund, Cathrine Elisabeth Olsen, Judith Jing Wen Wong, Petter Angell Olsen, Nina Therese Solberg, Anders Høgset, Stefan Krauss, Pål Kristian Selbo

**Affiliations:** 1 Unit for Cell Signaling, Institute of Microbiology, Rikshospitalet, 0372 Oslo, Norway; 2 Hybrid Technology Hub - Centre of Excellence, Institute of Basic Medical Sciences, Faculty of Medicine, University of Oslo, PO Box 1112, Blindern, 0317 Oslo, Norway; 3 Department of Radiation Biology, Institute for Cancer Research, Norwegian Radium Hospital, Oslo University Hospital, 0379 Oslo, Norway; 4 PCI Biotech AS, Ullernchaussèn 64, 0379 Oslo, Norway

**Keywords:** CD105, Endoglin, Pancreatic cancer, Photochemical internalization, Autophagy, 5-FU resistance

## Abstract

**Background:**

Development of resistance to 5-fluorouracil (5-FU) is a major problem in treatment of various cancers including pancreatic cancer. In this study, we reveal important resistance mechanisms and photochemical strategies to overcome 5-FU resistance in pancreatic adenocarcinoma.

**Methods:**

5-FU resistant (5-FUR), epithelial-to-mesenchymal-like sub-clones of the wild type pancreatic cancer cell line Panc03.27 were previously generated in our lab. We investigated the cytotoxic effect of the endosomal/lysosomal-localizing photosensitizer TPCS_2a_ (fimaporfin) combined with light (photochemical treatment, PCT) using MTS viability assay, and used fluorescence microscopy to show localization of TPCS_2a_ and to investigate the effect of photodamage of lysosomes. Flow cytometric analysis was performed to investigate uptake of photosensitizer and to assess intracellular ROS levels. Expression and localization of LAMP1 was assessed using RT-qPCR, western blotting, and structured illumination microscopy. MTS viability assay was used to assess the effect of combinations of 5-FU, chloroquine (CQ), and photochemical treatment. Expression of CD105 was investigated using RT-qPCR, western blotting, flow cytometry, and fluorescence microscopy, and co-localization of TPCS_2a_ and anti-CD105-saporin was assessed using microscopy. Lastly, the MTS assay was used to investigate cytotoxic effects of photochemical internalization (PCI) of the anti-CD105-immunotoxin.

**Results:**

The 5-FUR cell lines display hypersensitivity to PCT, which was linked to increased uptake of TPCS_2a_, altered lysosomal distribution, lysosomal photodamage and increased expression of the lysosomal marker LAMP-1 in the 5-FUR cells. We show that inhibition of autophagy induced by either chloroquine or lysosomal photodamage increases the sensitivity to 5-FU in the resistant cells. The three 5-FUR sub-clones overexpress Endoglin (CD105). Treatment with the immunotoxin anti-CD105-saporin alone significantly reduced the viability of the CD105-expressing 5-FUR cells, whereas little effect was seen in the CD105-negative non-resistant parental cancer cell lines. Strikingly, using the intracellular drug delivery method photochemical internalization (PCI) by combining light-controlled activation of the TPCS_2a_ with nanomolar levels of CD105-saporin resulted in strong cytotoxic effects in the 5-FUR cell population.

**Conclusion:**

Our findings suggested that autophagy is an important resistance mechanism against the chemotherapeutic drug 5-FU in pancreatic cancer cells, and that inhibition of the autophagy process, either by CQ or lysosomal photodamage, can contribute to increased sensitivity to 5-FU. For the first time, we demonstrate the promise of PCI-based targeting of CD105 in site-specific elimination of 5-FU resistant pancreatic cancer cells in vitro. In conclusion, PCI-based targeting of CD105 may represent a potent anticancer strategy and should be further evaluated in pre-clinical models.

**Electronic supplementary material:**

The online version of this article (10.1186/s13046-017-0662-6) contains supplementary material, which is available to authorized users.

## Background

5-Fluorouracil (5-FU) is one of the standard chemotherapy drugs used to treat pancreatic cancer. However, treatment only extends survival modestly, and disease recurrence is typical due to drug resistance. We have previously established and in detail characterized 5-FU resistant (5-FUR) monoclonal cell lines from the pancreatic adenocarcinoma Panc03.27 cell line by long-term exposure to increasing doses of 5-FU. The 5-FUR cell lines showed alterations typical for epithelial–mesenchymal transition (EMT), including increased invasiveness, upregulation of mesenchymal markers (vimentin and N-cadherin), increased expression of the EMT-related membrane protein L1CAM, and downregulation of epithelial markers, e.g. E-cadherin and cytokeratin 19 [1]. Other mesenchymal-like phenotypes of the 5-FU-resistant clones observed were cell scattering and increased formation of pseudopodia, while the sensitive clones displayed a tightly packed epithelial morphology.

Photochemical internalization (PCI) is a drug delivery technology for local and light controlled cytosolic release of therapeutics entrapped in endosomes and lysosomes, confined in the illuminated area only. It is based on the use of an amphiphilic photosensitizer which anchors into the membrane of endo−/lysosomal compartments. Hence, drugs that are entrapped in such vesicles can, upon photosensitizer activation, be released into the cytosol due to membrane permeabilization induced by the generation of reactive oxygen species (ROS). Light-triggered activation of the PCI photosensitizer results in generation of mainly singlet oxygen, but also other ROS that may induce lipid peroxidation [2, 3]. The PCI principle has been documented in several animal models [4, 5] and for clinical evaluation the PCI-photosensitizer, TPCS_2a_ (fimaporfin) has been developed [6]. Recently, a Phase I dose-escalating trial of PCI of bleomycin was completed, a showing high degree of safety and tolerability [7]. Furthermore, PCI in combination with gemcitabine for the treatment of inoperable bile duct cancer (cholangiocarcinoma) was found safe in a Phase I trial [NCT01900158], and has been granted an orphan drug designation by the European Commission (EMA/COMP/513113/2016) and the U.S. FDA. Pancreatic adenocarcinoma is the most lethal of the common cancers, with a high unmet need for better treatment options. Surgery is the only curative therapy for pancreatic cancer (Stage 1 or II); yet only 15–20% of these patients are candidates for surgical resection [8]. Different regimens with chemotherapy have been evaluated; however, development of chemotherapy resistance is a major obstacle and a reason for pancreatic tumour recurrence. Photodynamic therapy (PDT) has been shown to be feasible, safe and to induce necrosis in locally advanced pancreatic cancer [9, 10], which suggests that the PCI technology should also be evaluated as a treatment strategy for this devastating disease.

In this work, we show that 5-FUR pancreatic cancer cells are hypersensitive to PCT (TPCS_2a_ + light). Our goal was to identify possible mechanisms behind the observed hypersensitivity, and investigate whether these mechanisms were related to alterations in the 5-FUR cells that occurred during the acquisition of 5-FU resistance. The increased sensitivity to PCT was linked to increased uptake of photosensitizer, altered lysosomal distribution, and increased expression of the lysosomal marker LAMP-1 in the 5-FUR cells. We show that inhibition of autophagy using chloroquine increases the sensitivity to 5-FU in the 5-FUR cells, indicating that acquisition of chemoresistance can be linked to alterations in the autophagy process in these cells. Finally, PCT resulting in lysosomal inactivation re-sensitized the chemoresistant pancreatic cancer cells to 5-FU treatment, which put forward PCI as a powerful anticancer strategy for this devastating cancer type.

CD105 (Endoglin) is a cell-surface glycoprotein that binds with high affinity to transforming growth factors (TGF) β1 and β3 [11] which are involved in regulation of cell differentiation and proliferation in most cell types. Elevated levels of CD105 expression is associated with human microvascular endothelium [12] and vascular endothelial cells in tissues undergoing active angiogenesis, such as regenerating and inflamed tissues or tumours [13]. The expression of CD105 on tumour-associated blood vessels makes CD105 an interesting target for therapy, as demonstrated by TRC105, the first humanized CD105-targeting antibody used in clinical trials for treatment of advanced or metastatic tumours [14]. Currently, TRC105 is being investigated in multiple clinical trials in combination with other agents [15–17]. Interestingly, CD105-expressing pancreatic- and breast cancer cells have been reported to show phenotypical alterations typical for an EMT, including increased migratory activity [18, 19].

By analyzing the published microarray data from the 5-FUR cell line B1V and the 5-FU sensitive (5-FUS) cell line Nt we found that CD105 mRNA was ~4.9-fold upregulated in B1V compared to in Nt. Based on this and the fact that CD105 is a druggable target, we hypothesized that PCI-controlled drug delivery of the immunotoxin anti-CD105-saporin would be an efficient and specific strategy to target and kill these 5-FU resistant pancreatic cancer cell lines. Here we show for the first time PCI-based targeting of CD105 as a promising strategy to target and kill CD105-overexpressing 5-FU resistant pancreatic cancer cells.

## Methods

### Cells and culture conditions

Panc03.27 pancreatic adenocarcinoma cells were obtained from ATCC (CRL-2549) and cultured in RPMI medium (R8758, Sigma-Aldrich) containing 10% FBS (16000–044, Invitrogen) and penicillin/streptomycin (17-603E, BioWhittaker) at 37 °C in a humidified atmosphere of 5% CO_2_. Cell lines were detached using Trypsin-EDTA (T3924, Sigma-Aldrich). Panc03.27 5-FU resistant cell lines were generated as previously described [1]. Cells were maintained in 1 μg/ml 5-FU, but were grown without 5-FU for 48 h prior to all experiments involving TPCS_2a_ and light treatment. The two 5-FU sensitive control cell lines (5-FUS) included in this work are named Panc03.27S–Nt (Nt) and Panc03.27S–Nw (Nw), while the three 5-FU resistant cell lines (5-FUR) are named Panc03.27R–B1L (B1L), Panc03.27R–B1Q (B1Q), and Panc03.27R–B1V (B1V).

### Drugs and chemicals

The photosensitizer meso-tetraphenyl chlorine disulfonate (TPCS_2a_ /Fimaporfin) was provided by PCI Biotech (Oslo, Norway). TPCS_2a_ was diluted in 3% polysorbate 80, 2.8% mannitol and 50 nM Tris (pH 8.5) to a final concentration of 0.35 mg/ml and kept at 4 °C protected from light. All work with TPCS_2a_ was performed under subdued light. The immunotoxin CD105-saporin (Beta-015) conjugate (CD105-SAP) was a gift from Advanced Targeting Systems. CD105-SAP is a chemical 270 kDa-conjugate between a mouse monoclonal antibody (clone 43A3) to human CD105 and the ribosome-inactivating protein saporin. Lysotracker® Green (pH 5.2) (L7526, Invitrogen) was used as a marker of acidic vesicles in the live microscopy studies. Chloroquine (CQ) (#14774) was purchased from Cell Signaling, and BSO (DL-Buthionine-(*S*,*R*)-sulfoximine, B2640) was purchased from Sigma.

### Light source

Illuminations of the cells were performed by using the Lumisource® (PCI Biotech) lamp. This light source consists of four standard light tubes (18 W/tube, OSRAM L 18/67), which emit broad band blue light with a main peak at approximately 435 nm with an output of ~13.5 mW/cm^2^ (1 min light exposure = 0.81 J/cm^2^) The irradiance varied less than 10% across the illumination area (765 cm^2^).

### Microarray hybridization and analysis

Microarray hybridization and analysis referred to in this manuscript (comparison of the Panc03.27S–Nt line and the Panc03.27R–B1V line) has been previously described [1] Microarray data can be found in GEOarchive (www.ncbi.nlm.nih.gov/geo/), accession number GSE58386.

### Photochemical internalization/photochemical treatment and viability assay

Three thousand cells per well were seeded in 96-well plates and allowed to attach overnight. The cells were incubated with the immunotoxin CD105-saporin (2.4 nM) or saporin as a control (6.48 nM, saporin was added in a molecular ratio of 2.7:1 to the immunotoxin (as determined and recommended by the producer) giving an equal ratio of saporin between the toxin and the immunotoxin), with or without the photosensitizer TPCS_2a_ (0.35 μg/ml) for 18 h. All treatments are summarized in a table in Fig. 8. Following treatment, cells were washed twice with drug-free culture medium, before they were incubated in drug-free culture medium and chased for 4 h. Cells were subsequently exposed to light, as described below, from 0 and up to 240 s. The viability of the cells was determined by performing an MTS assay 72 h after treatment, according to manufacturer’s protocol (CellTiter 96® AQ_ueous_ Non-Radioactive Cell Proliferation Assay, Promega), and absorbance at 490 nm was measured using a Victor Wallac 1420 Multilabel counter plate reader. Wells without cells incubated with MTS solution were used for background subtraction. When PCT was performed together with BSO, CQ, or 5-FU, the drugs were added at the same time as TPCS_2a_, and re-added after removal of the photosensitizer.

### Cellular accumulation of TPCS_2a_

Cells were seeded in 6-well plates and incubated with 0.35 μg/ml TPCS_2a_ at increasing times, including an additional 4 h drug free incubation time for cells incubated for 18 h, to mimic the PCI protocol. Cells were detached with trypsin and analyzed by flow cytometry as described below. Relative endocytosis and exocytosis rate was calculated based on the relative accumulation rate normalized to Nt cells when TPCS_2a_ was present in the media, while exocytosis rate is based on relative accumulation rate normalized to Nt cells when TPCS_2a_ was removed from the media (4 h).

### Intracellular ROS detection

Cells were seeded in 6 well plates and subjected to PCT (incubation with 0.35 μg/ml TPCS_2a_, as described in Methods *)*. Immediately after PCT, the cells were detached by trypsination, and untreated control cells were added for internal sample control. Then the cells were incubated with 0.1 mM 2′,7′-Dichlorofluorescin diacetate (DCFH-DA) (D6883, Sigma-Aldrich) for 1 h at 37 °C in the dark prior to flow cytometry.

### Measurements of cell size

Cells detached by trypsination were counted by a Coulter Counter Z2 (Beckman Coulter, CA, USA). The average median diameter was calculated from three individual experiments, of which 10,000 cells were counted per experiment within the diameter range of 8–24 μm. Dead cells were discriminated based on size. Calculations of surface area were performed using circle-sphere calculations.

### Flow cytometry

**Fig. 1:**
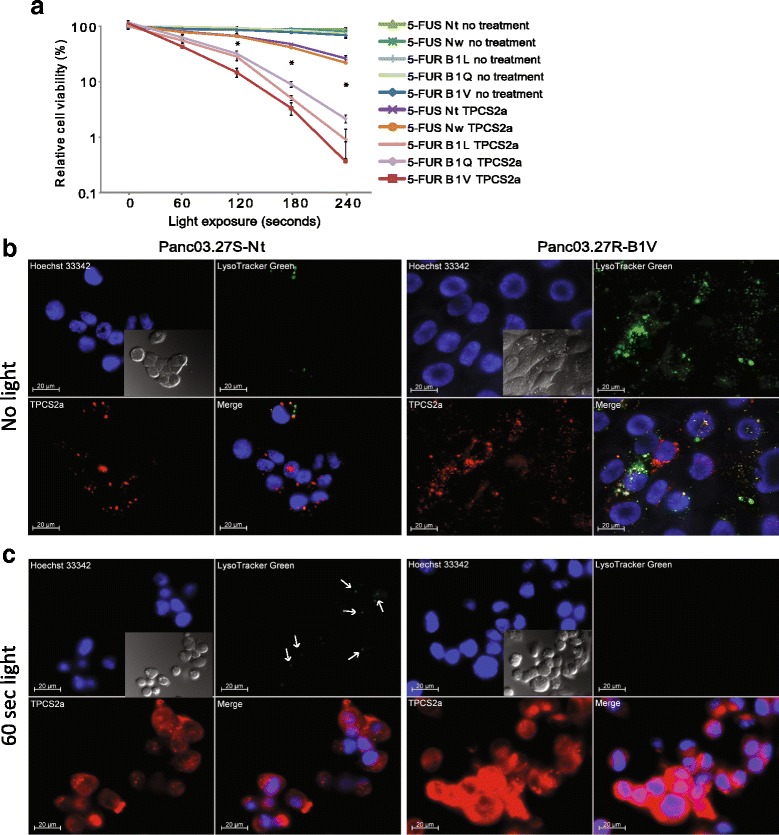
5-FU resistant pancreatic cancer cells are hypersensitive to PCT. **a** All cell lines were treated with PCT (TPCS_2a_ + light) with increasing light exposure up to 240 s. Reduction in cell viability (%) relative to untreated cells was measured 72 h after light exposure by MTS assay. The experiments were repeated more than five times, representative data are shown. Error bars represent standard deviation. Statistically significant difference between the effect of PCT in the 5-FU sensitive vs the 5-FU resistant cell lines (*P* < 0.01) is indicated by *. **b** and **c** Cellular uptake of TPCS_2a_ (red) and Lysotracker (LTG, green) in Panc03.27S–Nt and Panc03.27R–B1V cells, following **b** no light activation, and **c** 60 s light activation (white arrows are included to show remaining LTG signal in Nt cells). Co-localization of TPCS_2a_ (red) and LTG is indicated by yellow fluorescence. 10 μg/ml Hoechst 33342 and 50 nM LTG was added 15 min and 30 min prior to image acquisition, respectively. The scale bar is 20 μm

Cells were filtered through a 5 ml round-bottom tube with a cell strainer cap (Becton-Dickinson) and then analyzed by flow cytometry, performed by using a BD LSRII Flow Cytometer (Becton-Dickinson). Sample cells were distinguished from untreated control cells by detection of TPCS_2a_ fluorescence in the ROS study. Data were processed by the FlowJo version 7.6.5 software (Treestar, OR), as recommended by the Flow Cytometry Core Facility (Institute for Cancer Research, Oslo University Hospital). Dead cells were discriminated based on forward and side scatter parameters, and PI staining (1 μg/ml added immediately before analysis) detected by excitation by a 561 nm (40 mW) laser and collected through a 610/20 nm bandpass filter combined with a 600 nm dichroic longpass filter. TPCS_2a_ was excited by a 407 nm (100 mW) laser and collected through a 660/20 nm bandpass filter combined with a 635 nm longpass dichroic filter. DCF fluorescence was detected by excitation with a 488 nm (50 mW) laser, and collected through a 525/50 nm or a 530/30 nm emission filter combined with a 505 nm longpass dichroic filter. For flow cytometric analysis of CD105 expression in Fig. 1, an Attune acoustic focusing cytometer from Applied Biosciences was used for detection of the anti-CD105-Alexa488 antibody (323,209, Biolegend).

### Fluorescence microscopy

The cell lines Panc03.27S–Nt and Panc03.27R–B1V were used in fluorescence microscopy study of TPCS_2a_ and AlexaFluor488 conjugated anti-CD105 antibody (323,209, Biolegend). Thirty thousand cells per well were seeded on coverslips in 48-well plates (Nunc) and allowed to attach overnight. Anti-CD105-Alexa488 (1:1000) and/or 0.35 μg/ml TPCS_2a_ was added to the wells, and incubated for 18 h, washed twice with PBS, and incubated in drug free culture medium for 4 h (chase). Hoechst 33,342 (10 μg/ml) (H3570, Invitrogen) and 50 nM LysoTracker Green DND-26 (L7526, ThermoFisher Scientific) was added 15 min prior to image acquisition. For the fluorescence microscopy of TPCS_2a_ and 10 kDa AlexaFluor488-Dextran (D-22910, Invitrogen) 50,000 cells were seeded out as described above. Cells were then incubated with 0.4 μg/ml AlexaFluor488-Dextran and 1.4 μg/ml TPCS_2a_ over-night and subsequently washed and chased in drug-free medium for 1, 2 and 4 h, of which for each time points cells were assessed for fluorescent signals. Fluorescence images were acquired with a Zeiss Axioplan epifluorescence and phase-contrast microscope using a 63× oil immersion objective (Carl Zeiss AG, Oberkochen, Germany). A 450–490 nm band-pass excitation filter, 495 nm dichroic mirror, and a 500–550 band-pass emission filter were used for the measurement of the Alexa488-Dextran. Fluorescence from TPCS_2a_ was recorded using a 395–440 nm excitation filter, a beam splitter at 460 nm, and a 620 nm long pass filter. The fluorescence was detected with an AxioCamMR3 camera (Carl Zeiss). The software program AxioVision Analysis (Carl Zeiss) was used to process the images.

### LAMP1 and LC3B immunofluorescent staining and widefield fluorescence imaging using structured illumination microscopy (SIM)

Cells grown on coverslips pre-coated with poly-L-lysine (Sigma-Aldrich) were treated with TPCS_2a_ and light as described in Methods. Chloroquine treatment was performed by incubating the cells in the presence of 50 μM chloroquine for 24 h. For LAMP1 and LC3B immunostaining cells were fixed in ice-cold methanol for 5 min followed by incubation at with primary and secondary antibodies diluted in PBS with 4% bovine serum albumin (at room temperature, 1 h each). Nuclear counterstaining was performed with DAPI (1 μg/ml, 5 min at room temperature). The coverslips were mounted in ProLong Diamond Antifade Mountant (Thermo Fisher Scientific). The following primary antibodies and dilutions were used: LAMP1 (D2D11) (1:200, #9091, Cell Signaling Technology), LAMP1 (CD107A) (H4A3) (1:500, BD555798; BD Biosciences), LC3B (D11) (1:200, #3868, Cell Signaling Technology). Secondary antibodies (all from Thermo Fisher Scientific; 1:500): anti-Rabbit IgG Alexa488 (A-21206), anti-Mouse IgG Alexa488 (A-11001), anti-Rabbit IgG Alexa647 (A-21246). Fluorescent images were acquired with a Zeiss Elyra PS1 microscope system using standard filters sets and laser lines with a Plan-APOCHROMAT 63 × 1.4 NA oil objective. Images were acquired using the “Laser wide field (WF)” or structured illumination microscopy (SIM) mode of the system as indicated in the figure legend. Laser WF images (Fig. 7f) were acquired for 30 Z planes with a Z spacing of 0.08 nm between planes. Laser WF images displayed are maximum intensity projections rendered from all Z planes. SIM images (Fig. 4c) were acquired using 5 grid rotations with the 0.51 μm grid for 20 Z planes with a Z spacing of 0.11 nm between planes. SIM images were reconstructed with the following “Method” parameters in the ZEN black software (Carl Zeiss MicroImaging): Processing: manual; Noise Filter: −5.5; SR Frequency Weighting: 1; Baseline Cut; Sectioning: 100/83/83; Output: SR-SIM; PSF: Theoretical. Unless otherwise mentioned SIM images are displayed as maximum intensity projections rendered from all Z planes.

### Western blot

Cell extracts were made by adding cold RIPA buffer (Thermo Fisher Scientific) containing protease inhibitors (Protease Inhibitor Cocktail Tablets, Roche) and phosphatase inhibitors (PhosStop Tablets, Sigma-Aldrich) to cell plates after wash with cold PBS, following manufacturer’s protocol for preparation of cell extracts from adherent cells. Protein concentration was determined using the Pierce™ BCA protein Assay Kit (Thermo Fisher Scientific). 15 μg of protein was loaded on to gels (Novex™ Bis-Tris gels (3–20% and 4–12%) or Tris-Acetate gels (3–8%), Life Technologies) together with PageRuler pre-stained protein ladder (Fermentas) and analyzed with a Novex electrophoresis chambers (Life technologies). Proteins were transferred to 0.2 μm nitrocellulose membranes (Novex, Life Technologies), blocked with 5% milk (AppliChem) in 0.05% tween-20 in TBS (Medicago) for 1 h, and stained with primary (4 °C overnight with rocking in 5% milk, 0.05% tween-20 in TBS) and secondary antibodies (1 h at room temperature with rocking in 5% milk, 0.05% tween-20 in TBS). Bands were visualized using ECL™ Prime Western Blotting Detection Reagent (GE Healthcare) in a ChemiDoc™ Touch Imaging system (Bio-rad) developer. Image Lab™ Software (Bio-rad) was used to quantify bands (normalized against loading controls). Primary antibodies used; Actin (1:2000; A5441, Sigma-Aldrich), β-Tubulin III (1:1000; T2200, Sigma-Aldrich), CD105/Endoglin (1:1000, ab169545, Abcam), LC3B (1:1000; #2775, Cell Signaling), LC3B (D11) (1:1000, #3868, Cell Signaling Technology), LAMP1 (1:1000; #9091, Cell Signaling), SOD1 (1:200; #4266, Cell Signaling), SOD2 (1:500; #30080, Santa Cruz).

### RT-qPCR

Total RNA was isolated using the GeneElute miniprep kit (Sigma) following the manufacturer’s instructions. cDNA was synthesized using the SuperScript VILO kit (11,754,050, Life Technologies), and real-time PCR was carried out using TaqMan gene expression master mix (4,369,016, Life Technologies) according to the manufacturer’s instructions on a StepOnePlus cycler (Life Technologies, Waltham, Massachusetts, USA). GAPDH was used to normalize the amount of cDNA in each sample and to guarantee the comparability of the calculated mRNA expression in all samples analyzed. Data represent Means ± SD; *n* ≥ 3. In all real-time qPCR graphs, RNA quantity are relative to one of three biological replicates of the Nt sample, each biological replicate containing three technical replicates. Statistically significant difference between 5-FU sensitive and 5-FU resistant cell lines (*P* < 0.05) is indicated by*.

The following probes were used (all from Life Technologies):

LC3B; Hs00797944_s1, 4,453,320.

LC3A; Hs01076567_g1, 4,448,892.

SOD1; Hs00533490_m1, 4,453,320.

SOD2; Hs00167309_m1, 4,453,320.

LAMP1; Hs00931461_m1, 4,448,892.

CD105; Hs00923996_m1, 4,453,320.

### Statistical analysis

To assess whether the means of the different treatments results were significantly different we used the two-sided Student’s t-test. A minimum significance level of *P* < 0.05 was used for all statistical tests.

## Results

### Complete light-triggered elimination of lysosomes in 5-FU resistant pancreatic cancer cells

To investigate the effect of photochemical treatment (PCT; TPCS_2a_ + light) on the 5-FU resistant Panc03.27 cell lines B1L, B1Q, and B1V (5-FUR lines) compared to the 5-FU sensitive cell lines Nt and Nw (5-FUS lines) we exposed the cells to PCT with light treatments up to 240 s. The relative cell viability was after 180 s light treatment reduced by >90% in the 5-FUR lines, while the cell viability of the 5-FUS lines was still above 40% for Nt and Nw (Fig. 1a). After 240 s light treatment, the average relative cell viability of the 5-FUR lines was reduced to ~1%, while the average cell viability of the 5-FUS lines was still at ~25%. The cell viability for the 5-FU resistant lines was at all illumination times significantly reduced compared to the control lines.

We have previously documented that light-activation of TPCS_2a_ leads to spread of a diffuse fluorescence throughout the cytosol, indicating escape of photosensitizer from the endo/lysosomal compartments [20]. In Fig. 1b and c, the 5-FUS cell line Nt and the 5-FUR cell line B1V were exposed to TPCS_2a_ followed by 60 s light treatment (as in the PCT/PCI protocol), and LTG was added shortly before image acquisition. The LTG-stained vesicles (green puncta) seen before illumination (Fig. 1b) were completely eliminated in the PCT-treated B1V cells after illumination (Fig. 1c), indicating that light treatment leads to destruction of the acidic endosomes and lysosomes. In contrast, some LTG signals were still detected in the Nt cells after PCT at the same time point. Further, the lower images in Fig. 1c show a diffuse TPCS_2a_ fluorescence signal throughout the cell cytosol (of which the 5-FUR cells had a much stronger fluorescence intensity than the 5-FUS cells), in contrast to the sharp fluorescence puncta observed in the lower images in 1B, indicating escape of the photosensitizer as a result of endo/lysosomal membrane destruction.

### 5-FU resistant pancreatic cancer cells display both increased uptake of TPCS_2a_ and increased ROS production following PCT despite higher expression of SOD1 and SOD2

**Fig. 2:**
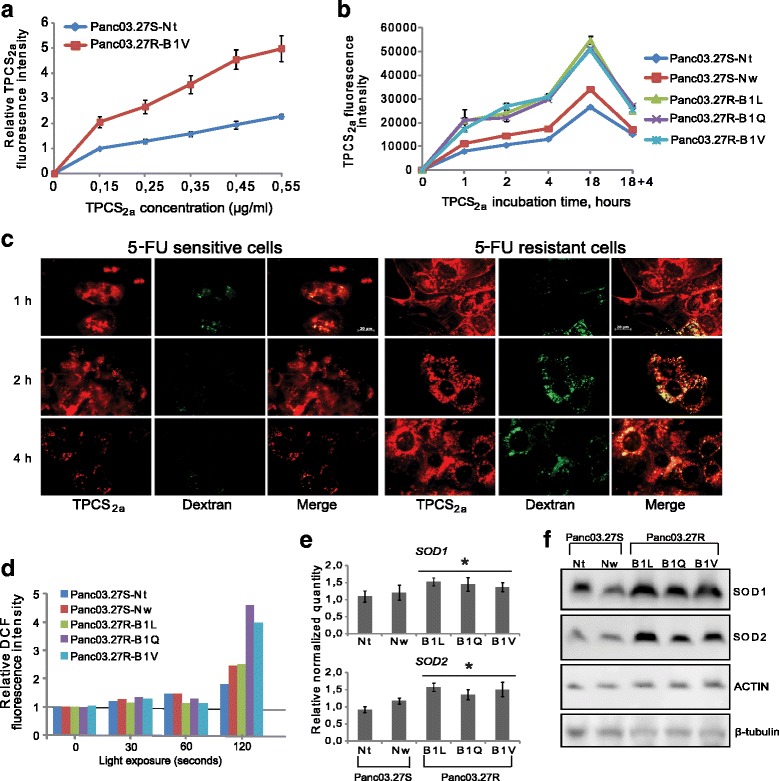
5-FU resistant pancreatic cancer cells display increased uptake and accumulation of TPCS_2a_, and show increased ROS production following PCT. TPCS_2a_ accumulation with **a** increasing TPCS_2a_ concentration and **b** increasing incubation time. The graphs show the median TPCS_2a_ fluorescence intensities in live cells and show the average of three and two individual experiments, respectively (Error bars = S.E.). **c** Representative epi-fluorescence microscopy images of live 5-FU sensitive and 5-FU resistant cells harvested 1, 2 and 4 h after TPCS_2a_ incubation. Co-stain of TPCS_2a_ and Alexa488-Dextran is indicated by yellow fluorescence in the rightmost images. The experiment was performed once but was also verified for the B1Q and the B1L cells (data not shown), confirming the data in (**a**) and (**b**). The scale bar is 20 μm. **d** Relative ROS generation measured 1 h after light exposure, with increasing PCT (TPCS_2a_ + light) illumination doses (30–120 s). The graph shows normalized median DCF fluorescence intensities representative of three individual experiments. **e** RNA levels (relative quantity) of *SOD1* and *SOD2* in all cell lines, as measured by RT-qPCR. Error bars represent standard deviation. Statistically significant difference between 5-FU sensitive and 5-FU resistant lines (*P* < 0.05) is indicated by *. **f** Western blot analysis showing SOD1 and SOD2 protein expression in untreated cells. The blot is representative from at least two individual experiments

We asked whether the increased sensitivity to PCT in the 5-FUR cell lines was caused by increased uptake of the photosensitizer in the cells at the moment of light induction (after 18 h incubation of TPCS_2a_ followed by wash of cells and a 4 h incubation in drug-free medium (PCI/PDT protocol)). To address this, we added increasing doses of TPCS_2a_ to the control line Nt and the 5-FUR cell line B1V, and measured relative TPCS_2a_ fluorescence intensity at the time of light induction (Fig. 2a). We observed an increase in fluorescence intensity with increasing doses of TPCS_2a_ in both cell lines. At 0.35 μg/ml, which is the concentration used in our PCT and PCI experiments, the 5-FUR line B1V accumulated 2.3-fold more TPCS_2a_ than the 5-FUS cell line Nt.

Next, we measured TPCS_2a_ fluorescence intensity in all cell lines at increasing incubation time points, including the time of light exposure in the PCT/PCI protocol (Fig. 2b). At 1 h incubation with TPCS_2a_, we observed a 2-fold higher uptake of TPCS_2a_ in the 5-FUR lines compared with the control lines, indicating an increased endocytosis rate in the 5-FUR cells (TPCS_2a_ is strongly amphiphilic and cannot penetrate through the plasma membrane). After 18 h, the average level of TPCS_2a_ in the 5-FUR lines was 1.7 times higher than in the average of the control lines Nt and Nw. Although the TPCS_2a_ levels decreased over time in all cell lines after washing, the 5-FUR lines had an average of 1.6-fold more TPCS_2a_ than the average of the control lines. As this is the time of light-triggered activation of TPCS_2a_ in our assays, it may partly explain the hypersensitivity of the 5-FUR lines to PCT (Fig. 1a).

The increased accumulation of TPCS_2a_ in the 5-FUR cells could be due to delayed exocytosis activity. To further investigate the endocytosis and exocytosis capacities of the 5-FUR and 5-FUS cell lines, we included the fluid-phase endocytosis marker Alexa488-Dextran (10 kDa) and combined it with TPCS_2a_ in fluorescence microscopy studies (Fig. 2c). Dextran is water soluble, does not bind to the plasma membrane and is taken up into cells by fluid phase endocytosis [21]. On the contrary, the strongly amphiphilic PCI-photosensitizer TPCS_2a_ is embedded in the lipid bilayer of the cell membrane, does not diffuse through the membrane and is hence taken up into cells by endocytosis. The sub-cellular localization of TPCS_2a_ prior to light activation is in endosomes and lysosomes [6, 22]. We monitored Alexa488-dextran and TPCS_2a_ uptake in the cells at different time points between wash (18 h) and time of light induction in our experiments (18 + 4 h). Images were taken after 1 h, 2 h, and 4 h chase in drug-free medium. The fluorescence images revealed cellular uptake of both TPCS_2a_ (red) and Alexa488-dextran (green), and a substantial co-localization (as observed by the yellow signal after merging the micrographs of the two dyes) was observed in the 5-FUR cells as compared to the 5-FUS cells. We detected higher signals of TPCS_2a_ and Alexa488-dextran in the 5-FUR cells after 1 h chase in drug-free medium. However, while a strong decrease in Alexa488-dextran- and TPCS_2a_ levels was seen from 1 h to 4 h after wash in the 5-FUS cells, we detected a delay in the excretion of both Alexa488-dextran and TPCS_2a_ in the 5-FUR cells. This could be a result of the higher endocytosis rate observed in the 5-FUR cells, as seen with TPCS_2a_ in Fig. 2a and b, but can also indicate a more rapid exocytosis in the 5-FUS lines than in the 5-FUR lines. It should however be mentioned that although we obtained higher levels of TPCS_2a_ at 18 + 4 h in the 5-FUR cells (Fig. 2b), we observed a similar decline in TPCS_2a_ levels from 18 h to 18 + 4 h in all cell lines. In the 5-FUS lines Nt and Nw the decline in TPCS_2a_ levels were reduced by 57 and 50%, respectively, while in the 5-FUR lines B1L, B1Q, and B1V the level was reduced by 45, 53, and 50%, respectively (Fig. 2b).

Cell surface area can influence the uptake of photosensitizers [23]. The 5-FUR cells appeared larger than the control cells when studied by microscopy. By using the Coulter Counter Z2, the mean diameter of the 5-FUR cells was calculated to be 22% larger than that of the mean of the control cell lines. Accordingly, the calculated cell surface area of the 5-FUR lines was 51% larger. TPCS_2a_ accumulation at 18 h was, hence, higher than one would expect in the 5-FUR cells. Thus, the increased accumulation of TPCS_2a_ in our 5-FUR lines could only partly be attributed to the increased cell size. At the PCI time point (18 h incubation and 4 h wash), however, photoactivable (fluorescing) TPCS_2a_ is only a factor of 1.6 higher in the 5-FUR cells. Taking into the account the cell surface area difference between the 5-FUS and the 5-FUR cells is only a factor of 1.5 in favor of the 5-FUR cells, the relative difference of the TPCS_2a_ signal is marginal. Yet, it seems that when TPSC_2a_ is released into the cytosol, the difference in photoactivable TPCS_2a_ is much higher in the 5-FUR cells indicating that a large fraction of TPCS_2a_ is stacked in the lysosomes, an thereby not photoactivable (stacking-induced fluorescence quenching in lysosomes), prior to PCT/PCI. Thus, the total accumulation of TPCS_2a_ is much higher in the 5-FUR cells.

Increased generation of ROS after PCT is a normal response to increased intracellular levels of photosensitizers [2]. Indeed, an increase in ROS production was detected in all cell lines subjected to PCT (after 120 s light exposure, Fig. 2d). The highest increase of ROS generation was detected in the 5-FUR lines B1Q and B1V (4.6-fold and 4.1-fold, respectively), whereas the control cell lines Nt and Nw displayed a 1.7-fold and 2.4-fold increase in ROS production, respectively. The increased generation of ROS in B1Q and B1V compared to Nt and Nw correlates well with the increased accumulation of TPCS_2a_ seen in Fig. 2a and b. In the 5-FUR line B1L, however, generation of ROS was near similar to the Nt and Nw cells at 120 s light exposure.

**Fig. 3:**
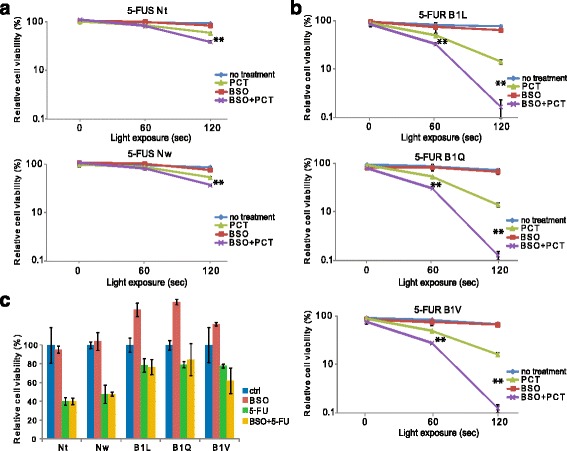
Co-treatment with BSO increases the cytotoxic effect of PCT, but not to 5-FU. **a** 5-FU sensitive (Nt, Nw) and **b** 5-FU resistant cell lines (B1L, B1Q, B1V) were subjected to co-treatment with BSO and PCT for 72 h after light. BSO (100 μM) was added together with TPCS_2a_ the day before, and BSO was added again after wash (total treatment time: 96 h). **c** All cell lines were subjected to co-treatment with 100 μM BSO and 1 μg/ml 5-FU for 72 h. Reduction in cell viability (%) relative to untreated cells was measured by MTS assay. The experiments were performed at least 3 times, representative data are shown. Error bars represent SD. Statistically significant difference between PCT and PCT + BSO in **a**) and **b**) is indicated by **. *P* < 0.001

5-FU treatment has been shown to induce ROS-generation in colon carcinoma cells [24]. We therefore wanted to investigate whether the 5-FUR cell lines expressed higher levels of ROS quenching enzymes as a result of long-term 5-FU treatment. Using RT-qPCR, we found the ROS scavenging enzymes superoxide dismutase (SOD) 1 and SOD2 RNA expression levels to be significantly higher in the 5-FUR cell lines compared to the control cell lines (Fig. 2e). These data correlate well with the previously published microarray analysis data comparing SOD1 and SOD2 levels in Nt and B1V (Additional file 1: Figure S1A and B). Further, we found increased protein levels of SOD 2 in the 5-FUR cell lines compared to the control lines (Fig. 2f). This increase in anti-ROS enzymes might be a protection mechanism developed in the 5-FU resistant cells in the process of acquiring resistance to 5-FU. Despite this fact, the 5-FUR cells were more sensitive to PCT than the 5-FUS cells. We therefore asked whether increased sensitivity in the 5-FUR cells could be explained by less quenching of PCT-induced ROS by GSH, thereby increasing the PCT-sensitivity of the 5-FU-resistant cell lines. Glutathione (GSH) is a key antioxidant in normal and cancer cells [25], and may neutralize ROS by donating either H^+^ or e^−^, in e.g. redox reactions catalyzed by glutathione peroxidases (GPx) and for the transport of xenobiotics by the GSH efflux pump [26]. High levels of GSH within tumor cells may, hence, reduce the ability of e.g. PCT to selectively kill cancer cells [27]. To test this, GSH depletion by buthionine sulfoximine (BSO), an inhibitor of gamma-glutamylcysteine synthetase, was used. Indeed, the combination of PCT and BSO induced a significant reduction in cell viability in all cell lines compared to PCT alone (Fig. 3a and b). In the 5-FUR lines B1L, B1Q and B1V, co-treatment with PCT and BSO followed by 120 s light treatment resulted in 9-fold, 11-fold, and 12-fold reduction of cell viability compared to PCT, respectively (Fig. 3b). In the 5-FUS lines Nt and Nw, co-treatment resulted in 1.5-fold and 1.4-fold reduction, respectively (Fig. 3a). Treatment with BSO alone had no effect on cell viability in any of the cell lines. Further, co-treatment with BSO did not increase sensitivity to 5-FU in either cell line, ruling out GSH as a protective mechanism against 5-FU (Fig. 3c).

### Altered expression and localization of the lysosomal marker LAMP-1 in 5-FU resistant cell lines

**Fig. 4:**
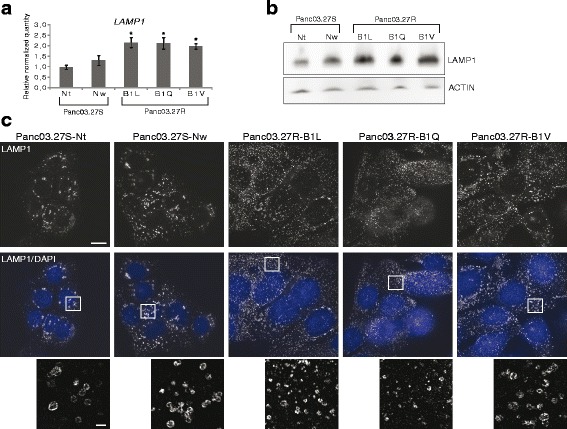
Altered expression and localization of the lysosomal marker LAMP1 in the 5-FUR cell lines. **a** RNA levels (relative quantity) of *LAMP1* in all cell lines, as measured by RT-qPCR. Error bars represent standard deviation. Statistically significant difference between 5-FU sensitive and 5-FU resistant lines (*P* < 0.05) is indicated by *. **b** All cell lines were subjected to immunoblotting to detect total levels of LAMP1. The experiment was repeated twice (*n* = 3), representative blots are shown. **c** All cell lines were subjected to super resolution microscopy (SIM) following LAMP1 and DAPI stain (as described in Methods). The experiment was repeated twice. The white scale bar on the top images is 10 μm, while the scale bar at the lower (enlarged) image is 1 μm

The lysosomal marker LAMP1 (Lysosome-associated membrane protein 1) is involved in regulating lysosomal motility during lysosome-phagosome fusion and cholesterol trafficking [28, 29]. The previously published mRNA expression data [1] showed a 2-fold increase in LAMP1 expression in the 5-FUR cell line B1V compared to the 5-FUS line Nt (Additional file 1: Figure S1C). Significantly increased RNA expression level of LAMP1 in the 5-FUR lines compared to the 5-FUS lines was confirmed using RT-qPCR (Fig. 4a). Results from Western blot analysis show that the level of the LAMP1 protein was indeed higher in the 5-FUR cell lines (Fig. 4b). When we investigated the subcellular localization of LAMP1 in 5-FUS and 5-FUR cell lines by widefield fluorescence imaging using structured illumination microscopy (SIM) we observed that the LAMP1 puncta appeared smaller and more numerous in the 5-FUR lines compared to the 5-FUS lines (Fig. 4c). The enlarged images (bottom) clearly showed the localization of LAMP1 in the membranes of circular vacuoles in the 5-FUS cell lines. Together, these data suggest an altered lysosomal biogenesis in the 5-FUR lines.

### Inhibition of autophagy re-sensitizes 5-FU resistant cells to 5-FU

Autophagy is a catabolic process for the autophagosomic-lysosomal degradation of bulk cytoplasmic contents [30, 31]. Increased autophagy has been reported as a survival mechanism in response to 5-FU treatment [32]. However, resistance to 5-FU has also been linked to decreased autophagic activity in other models [33], suggesting its role to be cell line dependent.

**Fig. 5:**
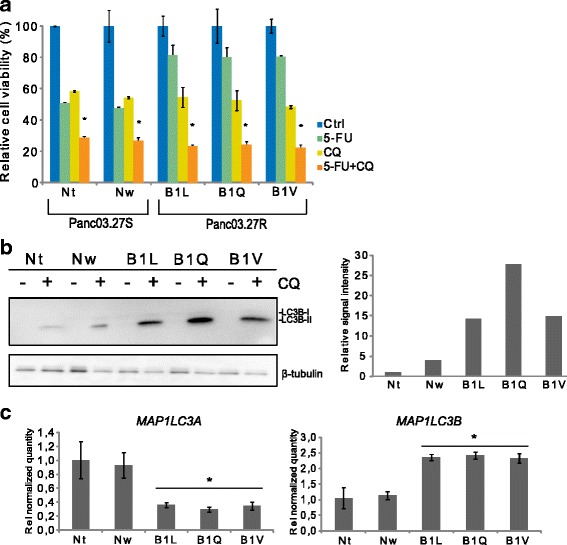
Inhibition of autophagy resensitizes resistant cells to 5-FU. **a** All cell lines were subjected to co-treatment with 5-FU (1 μg/ml) and chloroquine (CQ) (10 μM) for 72 h. Reduction in cell viability (%) relative to untreated cells was measured by MTS assay. Experiments were performed at least three times, representative data are shown. Error bars represent SD. Statistically significant difference between treatment with CQ alone and co-treatment (5-FU and CQ) is indicated by *. *P* < 0.01. **b** All cell lines were subjected to immunoblotting to detect total levels of LC3B-I and LC3B-II, following treatment with CQ (50 μM) for 24 h. The experiment was repeated twice. The intensity of bands from CQ-treated cells are quantified relative to loading control bands, with the intensity level in Nt set to 1. **c** RNA levels (relative quantity) of *MAP1LC3A* and *MAP1LC3B* in all cell lines, as measured by RT-qPCR. Error bars represent standard deviation. Statistically significant difference between 5-FU sensitive and 5-FU resistant lines (P < 0.05) is indicated by *

The increased number of lysosomes seen in the 5-FUR lines (Fig. 4c) made us speculate whether the process of autophagy was altered in these cells. To investigate this, we exposed the cells to 5-FU in the presence of the autophagy inhibitor chloroquine (CQ), which prevents autophagosomal fusion and degradation [34]. As shown in the viability assay in Fig. 5a, all cell lines displayed the same sensitivity to CQ, with ~40% reduction in relative cell viability after 72 h treatment. Further, our results showed a significant decrease in cell viability in all cell lines following co-treatment with 5-FU and CQ compared to treatment with either drug alone, and the viability was reduced to ~25% in all cell lines, regardless of 5-FU sensitivity. These data indicated that inhibition of autophagy increased the effect of 5-FU treatment, and suggests that autophagy is involved as resistance mechanism to 5-FU in the 5-FUR cells.

Then we investigated the effect of CQ treatment on the autophagy membrane marker LC3B, using Western blotting (Fig. 5b). Upon induction of autophagy, LC3 becomes conjugated to phosphatidylethanolamine (PE) in the autophagy membrane [35]. Lipidation of LC3 causes it to run at a lower molecular weight (LC3-II) than the cytosolic form (LC3-I) upon SDS-PAGE. LC3-II remains conjugated to the autophagosomal membranes during the autophagic process and is degraded upon fusion with lysosomes. CQ-mediated inhibition of these fusion events blocks LC3-II degradation [32]. Western blot results show that the protein level of LC3B is increased in all cell lines following CQ treatment. However, the intensity of the LC3B-II bands are three times stronger (B1L and B1V compared to Nw) or more in the 5-FUR cell lines following CQ treatment. Further, RT-qPCR results show that the RNA expression level of *MAP1LC3B* was twice as high in the 5-FUR lines compared to the 5-FUS lines, while *MAP1LC3A* levels were actually on average three times lower in the 5-FUR cells than in the 5-FUS lines (Fig. 5c). These data confirm microarray data for RNA expression level of *MAP1LC3A* and *MAP1LC3B* in the 5-FUS line Nt compared to the 5-FUR line B1V (Additional file 1: Figure S1C).

### PCT re-sensitizes cells to 5-FU

**Fig. 6:**
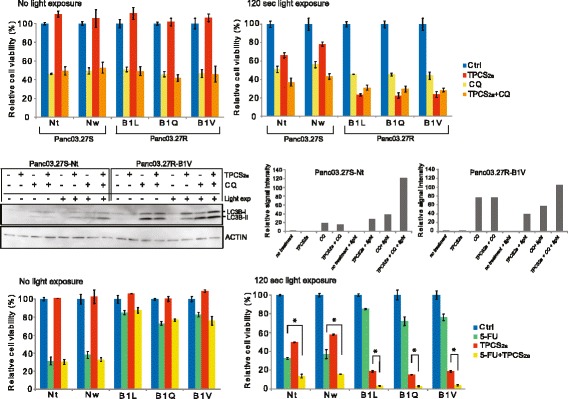
PCT sensitizes all cell lines to 5-FU. **a** All cell lines were subjected to co-treatment with TPCS_2a_ and chloroquine (CQ) (10 μM). CQ was added together with TPCS_2a_ the day before, and re-added after wash (total treatment time: 96 h). TPCS_2a_ and light treatment (PCT) was performed as described in the PCI protocol in section 2.5 in Methods. Graphs show relative cell viability (%) following no light exposure, and 120 s light exposure. Reduction in cell viability (%) relative to untreated cells was measured by MTS assay. **b** Left; All cell lines were subjected to immunoblotting to detect total levels of LC3B-I and LC3B-II following co-treatment with TPCS_2a_ and CQ (10 μM). CQ was added together with TPCS_2a_ the day before, and re-added after wash (total treatment time: 96 h). Right; Quantification of LC3B-II signal intensity relative to intensity of Actin signal. Signal intensity is normalized to no treatment for each cell line. **c** All cell lines were subjected to co-treatment with TPCS_2a_ and 5-FU (1 μg/ml). 5-FU was added together with TPCS_2a_ the day before, and re-added after wash (total treatment time: 96 h). Graphs show relative cell viability (%) following no light exposure, and 120 s light exposure. Reduction in cell viability (%) relative to untreated cells (Ctrl) was measured by MTS assay. Representative data are shown. Error bars represent SD. Statistically significant difference between PCT and PCT + 5-FU in the 5-FU resistant lines and statisitically significant difference between 5-FU treatment and PCT + 5-FU treatment in the 5-FU sensitive cell lines is indicated by *. *P* < 0.05

Photodamage of lysosomes may compromise the completion of the autophagic process. Therefore, even though autophagosomes form, digestion of their content is aborted because of the absence of functional lysosomes [36]. We investigated the effect of photochemical exposure (TPCS_2a_ ± 120 s light exposure) in the presence of CQ on cell viability in all cell lines (Fig. 6a). When we compared the effect PCT + CQ to the effect of PCT alone, we did not see any additional reduction in relative cell viability in the 5-FUR lines, rather, the cell viability seemed to be slightly higher with the co-treatment. In the 5-FUS lines, PCT combined with CQ lead to a ~14% reduction in cell viability compared to treatment with CQ alone.

Then we investigated the effect of co-treatment with PCT and CQ on the protein level of LC3B, using Western blotting (Fig. 6b). We observed an increase in LC3B-II signal intensity following PCT (TPCS_2a_ + light) in both cell lines compared to no treatment, but the increase in relative signal intensity observed in the 5-FUR line B1V was higher than in Nt (40-fold and 28-fold, respectively). Further, CQ treatment induced an increase in LC3B-II signal in both cell lines, regardless of light treatment, but the B1V line exhibited a higher increase in signal intensity than Nt (80-fold versus 19-fold). In support of this, fluorescence microscopy studies showed increase in LC3B fluorescence puncta following CQ treatment in both the 5-FUR line B1V and the 5-FUS line Nt, but the LC3B fluorescence signal appeared much stronger in the B1V line compared to the Nt line (Additional file 2: Figure S2). Further, in both cell lines, CQ treatment resulted in a 1.4-fold higher relative LC3B-II signal compared to the LC3B-II signal detected after PCT. When the cells were exposed to PCT in the presence of CQ, an additional increase in the LC3B-II signal was observed compared to the LC3B-II signal seen with CQ treatment + light. In the Nt line, the co-treatment resulted in a 3.2-fold increase in LC3B-II signal compared to treatment with CQ alone. In line with this, the co-treatment resulted in an additive effect on cell viability compared to treatment with CQ alone (~14% reduction, Fig. 6a). However, the increase in LC3B-II signal intensity seen with co-treatment in the B1V line was 1.8 fold, and was not accompanied by a reduction in cell viability with co-treatment compared to CQ treatment alone.

Then we investigated whether PCT-induced inhibition of autophagic flux increased the effect of 5-FU treatment, as seen with CQ-mediated inhibition of autophagy. After the cells were exposed to 5-FU during PCT (co-incubation with TPCS_2a_), a significant reduction of cell viability was seen after 120 s light exposure in the 5-FUR cells (Fig. 6c) compared to PCT alone. A significant reduction in cell viability was also seen in the 5-FU sensitive cell lines when co-treatment was compared to either treatment alone. The percentage reduction in cell viability when comparing PCT to co-treatment was larger in the 5-FU sensitive lines. However, the reduction in cell viability when comparing 5-FU treatment alone to 5-FU treatment combined with PCT was much higher in the 5-FU resistant cells. Altogether, these results indicate that autophagy is an important resistance mechanism in these cells, and that inhibition of the autophagic flux by photochemical targeting of lysosomes can contribute to increased sensitivity to 5-FU treatment.

### Increased expression of CD105 and specific cytotoxicity of CD105-saporin in 5-FU resistant cells

**Fig. 7:**
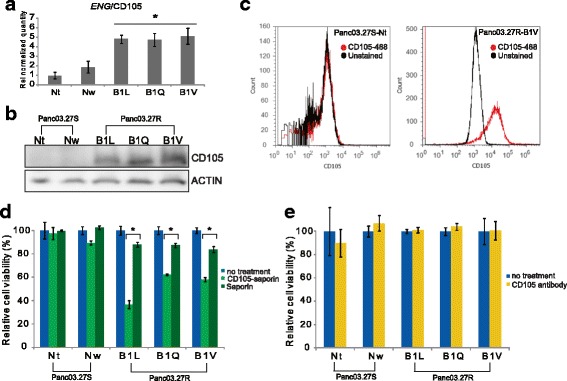
Increased expression of CD105 and selective binding of CD105-saporin in 5-FU resistant cells. **a** RNA levels (relative quantity) of CD105/*ENG* in all cell lines, as measured by RT-qPCR. Error bars represent standard deviation. Statistically significant difference between 5-FU sensitive and 5-FU resistant lines (P < 0.05) is indicated by *. **b** All cell lines were subjected to immunoblotting to detect total protein levels of CD105. **c** Flow cytometric analysis was used to detect membranous expression of CD105 in the 5-FU sensitive cell line Panc03.27S–Nt and the 5-FU resistant cell line Panc03.27R–B1V. **d** and **e** All cell lines were treated with CD105-saporin or saporin (**d**) and anti-CD105 antibody alone (**e**) for 72 h. Reduction in cell viability (%) relative to untreated cells was measured by MTS assay. The viability experiments were repeated at least 3 times, representative data are shown. Error bars represent SD. Statistically significant difference between treatment with CD105-saporin and saporin alone in (**d**) is indicated by *. P < 0.05

The endosomal/lysosomal localizing photosensitizer TPCS_2a_ (fimaporfin) is used in the drug delivery technology photochemical internalization (PCI) in combination with drugs that usually are trapped in endosomes and/or lysosomes. Hence, the photodamage of these organelles induces cytosolic release of the drug. Microarray data previously published [1] show upregulation of a number of druggable membrane proteins, including CD105 (endoglin), on the 5-FUR pancreatic cancer cell line Panc03.27R–B1V compared to the 5-FUS line Panc03.27S–Nt. We selected the membrane protein CD105 (endoglin) as a target candidate since the RNA level in B1V was found increased (4.9-fold) compared to the expression in the Nt line [1] (Fig. 7a). RT-qPCR and Western blotting experiments confirmed increased RNA expression level (Fig. 7a) and protein level (Fig. 7b) of CD105 in all 5-FUR cell lines compared to 5-FUS cell lines. This was further validated by flow cytometric detection of increased membranous CD105 expression on the B1V cell line compared to the Nt cell line (Fig. 7c).

As a proof-of-concept for cytotoxic PCI-based targeting of CD105 we decided to use an immunotoxin consisting of an antibody conjugated to the ribosomal inactivating protein saporin, CD105-saporin, and to investigate the specific cytotoxic effect of this conjugate on cell viability (MTS assay) in all cell lines. As seen in Fig. 7d, the effect on cell viability after 72 h treatment with CD105-saporin was significantly higher in the 5-FUR cell lines (40–60% reduction of cell viability) than in the 5-FUS lines. Notably, the 5-FUR cell lines were also slightly more sensitive to treatment with saporin alone (12, 13, and 16% reduction of cell viability for B1L, B1Q, and B1V, respectively) as compared to the control lines, which did not respond to saporin treatment. Treatment with naked CD105 antibody of the same clone (A43A3) that was used to produce the immunotoxin conjugate did not reduce cell viability (Fig. 7e), indicating that the cytotoxicity of the CD105-saporin was induced after receptor-mediated uptake.

### Efficient, specific and light controlled targeting of CD105-positive cancer cells by PCI of CD105-saporin

**Fig. 8:**
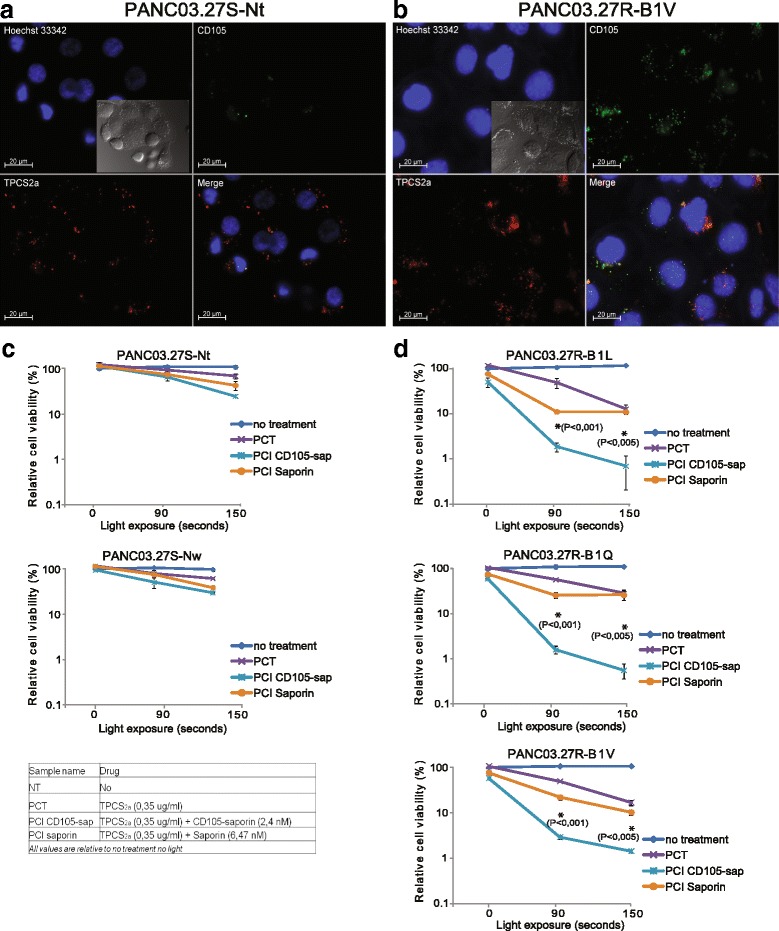
PCI-triggered endosomal escape of CD105-saporin induces specific and efficient cytotoxicity. **a** and **b** Cellular uptake of CD105–488 and TPCS_2a_ in **a** Panc03.27S–Nt and **b** Panc03.27R–B1V. Co-localization of TPCS_2a_ (red) and Lysotracker (green) is indicated by yellow fluorescence. 10 μg/ml Hoechst 33,342 and 50 nM LysoTracker were added 15 min and 30 min prior to image acquisition, respectively. The scale bar is 20 μm. **c** and **d** 5-FU sensitive (**c**) and 5-FU resistant (**d**) cell lines were subjected to either PCT, PCI of CD105-saporin, or PCI of saporin, following the PCI protocol described in Methods. Reduction in cell viability (%) relative to untreated cells was measured 72 h after light exposure, by MTS assay. All treatments are listed in the inserted table. The experiments were repeated at least 4 times, representative data are shown. Error bars represent SD. Statistically significant difference between PCI of CD105-saporin and PCI of saporin at the different time points is indicated by * and the indicated *P*-value

Fluorescence microscopy was used to verify the uptake of the CD105-targeting antibody and intracellular co-localization of the antibody with TPCS_2a_ (Fig. 8a and b). The cells were subjected to fluorescence microscopy imaging after 18 h of incubation with Alexa-488 labeled CD105 antibody and TPCS_2a_, followed by 4 h of incubation in drug-free medium (chase, to mimic the PCI protocol). The photosensitizer (red) was readily taken up by both the 5-FUS Nt and the 5-FUR B1V cells and localized in granular organelles, representing endosomes and lysosomes. Notably, the 5-FUR B1Vcells contained a higher number of TPCS_2a_-stained puncta that appeared to be of different sizes. The Alexa-488-labeled anti-CD105 antibody (green) was readily taken up in the B1V cells, and appeared as fluorescent puncta and co-localized with TPCS_2a_ (as shown in yellow in the merged images of TPCS_2a_ and Alexa488-CD105 fluorescence signals). Alexa488-CD105 fluorescence puncta signals could be detected in the Nt cells as well, however, the signals were much weaker as compared with the B1V cells.

We further investigated the effect on cell viability in all cell lines following TPCS_2a_-PCI of CD105-saporin, using illumination times from 0 to 150 s (Fig. 8c and d). An overview of the experiment with all treatments and concentrations is shown in the table in Fig. 8. As described above, a 72 h treatment with CD105-saporin alone reduced cell viability by more than 40% in the 5-FUR cell lines (Fig. 7c). Exposure of cells to TPCS_2a_ without illumination did not have any significant cytotoxic effect, but PCI of CD105-saporin, after only 18 h incubation of the immunotoxin, significantly and strongly reduced the viability of the 5-FUR lines compared to PCI of saporin after both 90 s and 150 s light treatment (Fig. 8d). After 150 s light treatment, PCI of CD105-saporin reduced the viability of the 5-FUR lines to ~1%, while viability following PCI of saporin at the same light dose was >10%. In comparison, the viability of the control cell lines Nt and Nw remained >25% after PCI of CD105-saporin, even at the highest light exposure (150 s). The 5-FUS lines showed significantly lower sensitivity to PCT at both 90 and 150 s light treatment when compared to the 5-FUR lines (Fig. 2c and d). At 150 s light treatment (PCT alone) the viability was reduced by 1.4-fold for both of the 5-FUS cell lines, while the viability of the 5-FUR lines B1L, B1Q, and B1V was reduced by 7.7-fold, 3.4-fold and 5.9-fold, respectively. Altogether, these in vitro experimental data validate CD105 as a drug target for PCI-enhanced efficacy and specificity of CD105-targeting antibody-drug/toxin conjugates. We show for the first time PCI-based targeting of CD105 as a promising strategy to target and kill CD105-overexpressing 5-FU resistant pancreatic cancer cells.

## Discussion

In this work we show that the 5-FU resistant pancreatic adenocarcinoma cells are hypersensitive to PCT (photosensitizer + light) as compared to the 5-FU sensitive control cells, despite a higher de novo expression of SOD1 and SOD2, both potent reactive oxygen (ROS) quenchers, and a higher GSH dependency in the 5-FU resistant cells. Higher sensitivity to photochemical therapy was linked to a higher cellular uptake of the PCI-photosensitizer TPCS_2a_ in the 5-FU resistant cells. This could partly be explained by increased cell surface area and increased endocytosis rate. An increase in cell surface area following long term drug treatment is an interesting phenomenon that also has been reported for other cell lines [37]. Of relevance to this, increase in cell size has also been attributed to EMT and activation of the mTOR pathway in cancer cells [38]. Further, recent work of Lubeseder-Martellato and co-workers shows that oncogenic KRAS increases fluid phase endocytosis of pancreatic preneoplastic cells [39]. Although the 5-FUR cells were highly sensitive to photochemical treatment, we show here that it is possible to make them, and partly the control cell lines, even more sensitive to PCT by inhibiting the anti-ROS mediator glutathione (GSH) by BSO-treatment. This indicates that quenching of singlet oxygen by GSH is an important cell survival mechanism in these cells, and hence, this observation does not support our hypothesis that less quenching of PCT-induced ROS by GSH leads to increased sensitivity to PCT in the 5-FUR cells. Of relevance to our work, others have reported increased levels of intracellular GSH in carcinoma cells that have acquired 5-FU resistance after long term exposure to 5-FU [40]. However, in the same report it was shown that co-treatment with 5-FU and BSO did not significantly reduce 5-FU resistance. In line with this, we did not detect enhanced 5-FU sensitivity when the Panc03.27 cell lines were we co-treated with BSO and 5-FU.

Zhitomirsky et al. [41] reported that cancer cells with an increased number of lysosomes were more resistant to lysosome-sequestered drugs, suggesting a model of drug-induced lysosome-mediated chemoresistance. The lysosomes in the 5-FU resistant lines used in our work appeared to be smaller and more numerous, and expression of the lysosomal marker LAMP1 was increased on both protein- and RNA level, suggesting a higher level of lysosomal biogenesis. An increased number of lysosomes observed in this study could be one of several cumulative steps of adaptation to 5-FU*,* although it has been suggested that the number of lysosomes is not important in the case of resistance to 5-FU [41]. Nevertheless, the lysosomal alterations seen in our 5-FU resistant cells, combined with a larger cell surface and higher fluid phase endocytosis rate, may contribute to a higher accumulation of TPCS_2a_ and, hence, partly explain the increased sensitivity to PCT. Further, photochemical exposure of these cells resulted in endosomal escape of TPCS_2a_ and a complete eradication of LysoTracker green (LTG) puncta, indicating enhanced photochemical targeting and destruction (photodamage) of lysosomes in 5-FU resistant cells, compared to the non-treated 5-FUS control cells (Nt) where some LTG puncta was still detected after PCT.

Of relevance to our work, there are reports indicating that autophagy might be involved in resistance to 5-FU in cancer cells [32, 42]. In this study we found that CQ-mediated inhibition of the autophagosome-lysosome fusion, shown by inhibition of LC3B degradation, re-sensitized the 5-FUR cells to 5-FU. Strikingly, we found that PCT-mediated disruption of lysosomes and the subsequent inhibition autophagosome-lysosome fusion re-sensitized the 5-FUR cells to 5-FU in the same manner as CQ treatment. In the 5-FUR cells, photochemical treatment together with CQ did not further enhance the cytotoxic response seen with CQ treatment alone, even though an increase in LC3B-II protein was observed following co-treatment with PCT and CQ compared to CQ treatment alone. This indicates that although PCT and CQ destroy or modify lysosomes by different mechanisms (photodamage versus increase of pH, respectively and thereby both treatments inhibit fusion of autophagosomes with lysosomes), the outcome of these individual treatments are an inhibition of the autophagic flux, which in turn seems to sensitize the 5-FUR cells to 5-FU.

The three 5-FU resistant cell lines used in this work were established by long-term exposure of the primary pancreatic adenocarcinoma cell line Panc 03.27 to 5-FU, and show alterations typical for an EMT [1]. Indeed, RNA expression analysis showed a 4.7-fold upregulation of the mesenchymal cell surface marker CD105 in the 5-FUR cells. It is likely that the increased expression of CD105 is linked to activation of the EMT program during the acquisition of 5-FU resistance [1] as CD105 is not only a marker of mesenchymal cells, but is also shown to be overexpressed on EMT-derived epithelial cells [18, 19, 43, 44]. Of relevance to our work, a link between overexpression of CD105, EMT, and resistance to 5-FU was recently reported in hepatocellular carcinoma stem-like cells, where 5-FU and epirubicin treatment generated CD105-positive cells with an activated EMT-program [45]. In this study, we show for the first time that PCI of the immunotoxin CD105-saporin selectively targets and induces strong cytotoxic responses in the CD105 overexpressing 5-FU resistant pancreatic cancer cells in vitro in a light dose-dependent manner. The cytotoxic response is due to the internalization and endo−/lysosomal release of the ribosome inhibiting protein (RIP) saporin of the endoglin/CD105-targeting toxin, demonstrated by its co-localization with TPCS_2a_ in acidic endosomes and lysosomes prior to illumination, and the endo−/lysosomal rupture upon light exposure.

## Conclusions

In conclusion, our data indicate that autophagy is an important resistance mechanism against 5-FU chemotherapy in pancreatic cancer cells, and that inhibition of the autophagy process, either by CQ or TPCS_2a_ + light-induced lysosomal photodamage, can contribute to increased sensitivity to 5-FU therapy.

Photodynamic therapy is under pre-clinical and clinical evaluation for non-resectable pancreatic cancer, and we think the present study is of high importance for further pre-clinical evaluations as we demonstrate that photochemical treatment, by light-activation of the lysosomal localizing PCI photosensitizer TPCS_2a_, destroys the autophagic flux and thereby re-sensitizes resistant cell lines to 5-FU treatment. We have also, for the first time, demonstrated the promise of PCI-based targeting of CD105 in pancreatic cancer cells. PCI-induced delivery of CD105-saporin can be utilized for the site-specific elimination of 5-FU resistant pancreas cancer cells. We also suggest that our PCI-based CD105 targeting approach may represent a novel strategy to perform simultaneous light-triggered destruction of both tumor cells and tumor vasculature that are CD105^+^. Accordingly, this novel approach should be further evaluated in preclinical models as a dual anti-angiogenesis and anti-tumor-cell strategy.

## Additional files

Additional file 1: Figure S1. RNA expression levels in Nt and B1V from previously published microarray data, presented as fold change, where Nt is set to 1. RNA expression levels of A) SOD1 and SOD2, B) LAMP1 C) MAP1LC3A and MAP1LC3B and D) CD105. (PDF 293 kb)

Additional file 2: Figure S2. Increased LC3B fluorescence signal in B1V following CQ treatment. Panc03.27S–Nt and Panc03.27R–B1V cells were treated with 50 μM CQ for 48 h before they were subjected to immunofluorescence detection of LC3B (red signal), nucleus stained with DAPI (Blue signal) as described in Methods. The scale bar is 10 μm. (PDF 2517 kb)

## Abbreviations

5-FU: 5-Fluorouracil
ATCC: American Type Culture Collection
BSO: DL-Buthionine-(*S*,*R*)-sulfoximine
CQ: Chloroquine
EMT: Epithelial-mesenchymal transition
LAMP1: Lysosome-associated membrane protein 1
LTG: Lysotracker green
PCI: Photochemical internalization
PCT: Photochemical treatment
RIP: Ribosome inhibiting protein
ROS: Reactive oxygen species
SIM: Structured Illumination Microscopy
SOD: Superoxide dismutase
TPCS_2a_: Disulfonated tetraphenyl chlorin
WB: Western blotting
WF: Wide Field
