# Supplementary figures and images for: 5-FU resistant EMT-like pancreatic cancer cells are hypersensitive to photochemical internalization of the novel endoglin-targeting immunotoxin CD105-saporin
Source: J Exp Clin Cancer Res. 2017 Dec 19;36:187. doi: 10.1186/s13046-017-0662-6 (PMC5738190; doi:10.1186/s13046-017-0662-6)

**A**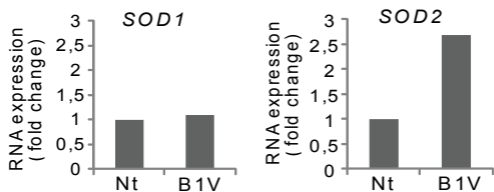**B**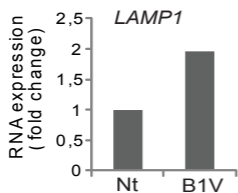**C**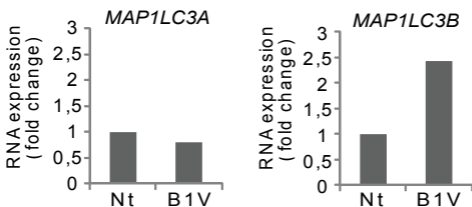**D**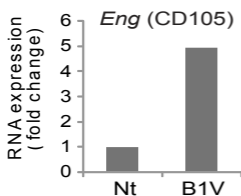

Supplement: Supplementary file 1 — RNA expression levels in Nt and B1V from previously published microarray data, presented as fold change, where Nt is set to 1. RNA expression levels of A) SOD1 and SOD2, B) LAMP1 C) MAP1LC3A and MAP1LC3B and D) CD105. (PDF 293 kb) [file 13046_2017_662_MOESM1_ESM.pdf]

## Panc03.27S-Nt

No treatment

CQ

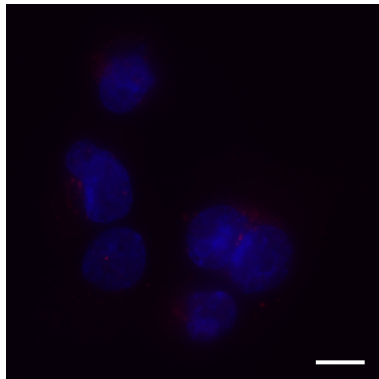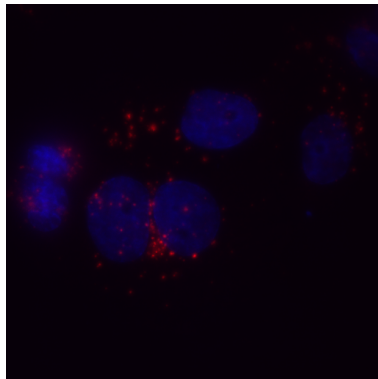

*LC3B* / *DAPI*

## Panc03.27R-B1V

No treatment

CQ

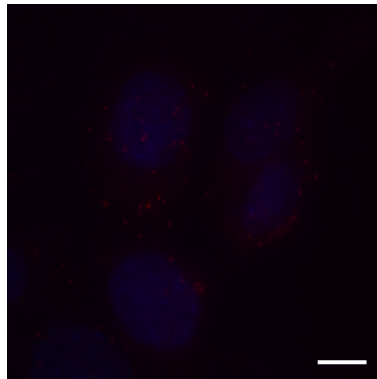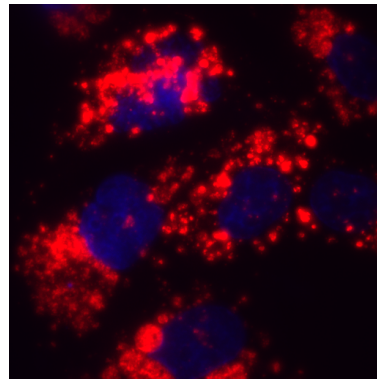

Supplement: Supplementary file 2 — Increased LC3B fluorescence signal in B1V following CQ treatment. Panc03.27S–Nt and Panc03.27R–B1V cells were treated with 50 μM CQ for 48 h before they were subjected to immunofluorescence detection of LC3B (red signal), nucleus stained with DAPI (Blue signal) as described in Methods. The scale bar is 10 μm. (PDF 2517 kb) [file 13046_2017_662_MOESM2_ESM.pdf]
